# Estimating excess mortality and economic burden of *Clostridioides difficile* infections and recurrences during 2015–2019: the RECUR Germany study

**DOI:** 10.1186/s12879-024-09422-w

**Published:** 2024-05-31

**Authors:** Ana Antunes, Aurore Tricotel, Adrian Wilk, Silvia Dombrowski, Hanna Rinta-Kokko, Fredrik L. Andersson, Subrata Ghosh

**Affiliations:** 1 IQVIA, Global Database Studies, Real World Solutions, Edifício 3, Lagoas Park, Oeiras, Lisboa, 2740 – 266 Portugal; 2 IQVIA, Real World Solutions, Paris, France; 3 Team Gesundheit, Gesellschaft für Gesundheitsmanagement mbH, Essen, Germany; 4 IQVIA, Real World Solutions, Frankfurt, Germany; 5 IQVIA, Global Database Studies, Real World Solutions, Espoo, Finland; 6 Ferring Pharmaceuticals, Copenhagen, Denmark; 7 College of Medicine and Health, University College Cork, Cork, Ireland

**Keywords:** *Clostridioides difficile*, Recurrences, Mortality, Healthcare costs, Germany

## Abstract

**Background:**

*Clostridioides difficile* infections (CDIs) and recurrences (rCDIs) remain a major public health challenge due to substantial mortality and associated costs. This study aims to generate real-world evidence on the mortality and economic burden of CDI in Germany using claims data between 2015 and 2019.

**Methods:**

A longitudinal and matched cohort study using retrospective data from Statutory Health Insurance (SHI) was conducted in Germany with the BKK database. Adults diagnosed with CDI in hospital and community settings between 2015 and 2018 were included in the study. Patients had a minimum follow-up of 12-months. All-cause mortality was described at 6-, 12-, and 24-months. Healthcare resource usage (HCRU) and associated costs were assessed at 12-months of follow-up. A cohort of non-CDI patients matched by demographic and clinical characteristics was used to assess excess mortality and incremental costs of HCRU. Up to three non-CDI patients were matched to each CDI patient.

**Results:**

A total of 9,977 CDI patients were included in the longitudinal cohort. All-cause mortality was 32%, 39% and 48% at 6-, 12-, and 24-months, respectively, with minor variations by number of rCDIs. When comparing matched CDI (*n* = 5,618) and non-CDI patients (*n* = 16,845), CDI patients had an excess mortality of 2.17, 1.35, and 0.94 deaths per 100 patient-months, respectively. HCRU and associated costs were consistently higher in CDI patients compared to non-CDI patients and increased with recurrences. Total mean and median HCRU cost per patient during follow-up was €12,893.56 and €6,050 in CDI patients, respectively, with hospitalisations representing the highest proportion of costs. A total mean incremental cost per patient of €4,101 was estimated in CDI patients compared to non-CDI patients, increasing to €13,291 in patients with ≥ 3 rCDIs.

**Conclusions:**

In this real-world study conducted in Germany, CDI was associated with increased risk of death and substantial costs to health systems due to higher HCRU, especially hospitalisations. HCRU and associated costs were exacerbated by rCDIs.

**Supplementary Information:**

The online version contains supplementary material available at 10.1186/s12879-024-09422-w.

## Background

*Clostridioides difficile* infection (CDI) is a common healthcare-associated infection and is increasingly acquired in community settings [1, 2]. A quarter of CDI patients develop at least one recurrence (rCDI) and the risk of rCDIs increases with each subsequent episode [3–8].

CDI can result in life-threatening inflammation of the colon and has been associated with substantial mortality [1, 2, 9, 10]. Studies conducted in North America and Europe using data from 2003 to 2016 reported 30-day mortality rates ranging between 11% and 18%, and 12-month mortality rates varying between 21% and 50% [11–16]. Patients with rCDIs experience a significantly higher risk of death when compared to patients without rCDIs [10, 12, 17]. Additionally, CDI poses a challenge to health systems due to long hospital stays, readmissions, and treatment costs, which increase among patients with rCDIs [18–20]. In 2012, the annual economic burden of CDI in the European Union was estimated at 3 billion euros and was predicted to almost double over the next four decades [21].

A systematic review and meta-analysis of studies and surveillance reports between 2005 and 2015 showed that Germany had one of the highest median overall incidence of CDI among European countries (7.00 CDI cases per 10,000 patient-days) [1]. However, a steady decline in the incidence of CDI in German hospitals has been observed since 2015 and attributed to the implementation of hygiene campaigns and strategies to tackle antibiotic resistance [22, 23]. Recent real-world data from hospital and community settings show a decline of 38% in the incidence rate of CDI between 2015 and 2019, which varied between 123.9 and 77.1 CDI episodes per 100,000 population, respectively [24].

Despite this downward trend, CDI remains a public health concern in Germany due to substantial morbidity, mortality, and associated costs. Moreover, rapidly increasing antibiotic-resistant strains of *C. difficile* pose additional challenges to CDI management [25]. While extensive literature is available on the mortality and economic burden of CDI, most studies focus on hospital settings and the United States (US) healthcare environment [10, 15, 26]. To address this gap, this study aimed to generate real-world evidence on the mortality and economic burden of CDI and rCDI using claims data from Germany.

## Methods

### Study design, data sources, and patient selection

The German RECUR study design has been described in a parallel publication (A. Tricotel, A. Antunes, A. Wilk, S. Dombrowski, H. Rinta-Kokko, F. L. Andersson, S. Ghosh, unpublished results). In brief, this observational retrospective cohort study was conducted using claims data from the BKK (*Betriebskrankenkassen*) database, which contains nationwide anonymized medical claims data for 5 million people with Statutory Health Insurance (SHI) representative of the German population regarding age and gender. Adult patients (≥ 18 years of age) identified with a diagnosis of CDI (International Classification of Diseases [ICD]-10 code: A04.7) recorded in a hospital or community setting between 2015 and 2019 were included. Detailed inclusion and exclusion criteria are listed in Table S1. The first CDI diagnosis was considered the index CDI episode. Index CDI episodes were classified according to setting of treatment (hospitalised CDI or community-treated CDI) and setting of infection (healthcare-, community-associated CDI or unknown) (Fig. S1). A rCDI episode was defined as a subsequent episode experienced within eight weeks from the start date of the last CDI episode (index CDI or previous rCDI) in alignment with the European Society of Clinical Microbiology and Infectious Diseases treatment guidance [27].

Analyses were restricted to patients with an index CDI episode between January 1, 2015 and December 31, 2018 and a minimum follow-up period of 12 months. Patients with a CDI episode within 6 months prior to index date were excluded. Follow-up period began from index date until death, loss to follow-up, or the end of the study period (December 31, 2019), whichever came first.

A matched cohort analysis was performed to assess excess mortality and incremental costs of healthcare resource utilization (HCRU), with a cohort of non-CDI patients selected based on an algorithm with the predefined criteria described below.

### CDI patients for matched cohort analysis

Based on the classification of index CDI episodes according to setting of treatment and infection, the following groups of patients were considered for the matched cohort analysis: healthcare-associated and hospitalised CDI (Group 1); community-associated and hospitalised CDI (Group 2); healthcare-associated and community-treated CDI (Group 3); and community-associated and community-treated CDI (Group 4). CDI patients reported with an unknown setting of infection were not selected for matching.

### Non-CDI patients for matched cohort analysis

A cohort of non-CDI patients was selected among patients from the BKK database without any record of CDI diagnosis, any biological test for the identification of bacterial toxins A or B, or any prescription of antibiotics indicated for CDI (non-topical metronidazole, vancomycin or fidaxomicin) during the study period or in the 12 months before the index date. Non-CDI patients were matched to each group of CDI patients based on the classification of index CDI episode date, age, gender, region, prior use of antibiotics (i.e., penicillin, cephalosporin, clindamycin, fluoroquinolones, macrolides, and rifaximin), Charlson Comorbidity Index (CCI) score, prior hospitalisation, and record of healthcare use. Table 1 details the specific criteria for matching. Up to three non-CDI patients were matched to each CDI patient. For non-CDI patients matched with hospitalised CDI patients (Groups 1 and 2), the start date of follow-up (i.e., index date) was set to the date of the hospitalisation closest to the index date of CDI patients. For those matched with community-treated CDI patients (Groups 3 and 4), the index date was set to the one of the corresponding CDI patient.

**Table 1:** Matching criteria for non-CDI patients for matched cohort analysis

	Non-CDI patients for matching			
Matching criteria	Healthcare-associated and hospitalised CDI patients (Group 1)	Community-associated and hospitalised CDI patients (Group 2)	Healthcare-associated and community-treated CDI patients (Group 3)	Community-associated and community-treated CDI patients (Group 4)
Age and gender	Same age (± 5 years) and sex as the matched CDI patient at index date			
Use of antibiotics	Prescription of any of the following antibiotics in the 12 months prior to index date: penicillin, cephalosporin, clindamycin, fluoroquinolone, macrolide, and rifaximin			
Comorbidities	Same age-adjusted CCI category: 0–6, 7–10, or ≥ 11 points			
Record of healthcare use	Record of a hospitalisation (not related to CDI) around the same date as the index date (± 1 month) as the matched CDI patient		Record of healthcare use (i.e. consultation, drug dispensation, biological tests) around the index date (± 1 month) as the matched CDI patient	
Region	Admission for hospitalisation in the same region as the matched CDI patient		Residents of the same region as the matched CDI patient at time of the healthcare use	
Prior hospitalisation	-	No hospitalisation within 3 months before the hospitalisation	Record of hospitalisation (not related to CDI) up to 1month preceding index date	No hospitalisation within 3 months before the hospitalisation

CCI, Charlson Comorbidity Index; CDI, *Clostridioides difficile* infection

### Outcomes

Outcomes of interest included all-cause mortality and excess mortality (6, 12, and 24 months after the index date); HCRU (i.e., hospitalisations, outpatient visits, pharmacological treatments, medical procedures, diagnosis tests, medical devices, and medical transportation), HCRU associated costs, and HCRU incremental costs. Costs were obtained from invoices for drug prescriptions in outpatient settings, medical aids, ambulatory care procedures and other services. Hospital stays costs were documented based on diagnosis-related groups. Since available billing data were linked to distinct patients, all costs were unambiguously assigned to individual patients.

### Data analysis

Data management and analysis were performed with SAS® version 9.4. For continuous variables, descriptive statistics were reported as mean, standard deviation (SD), median, 25th and 75th percentiles, and min and max. For categorical variables, absolute numbers and percentages were computed.

All-cause mortality at 6-, 12-, and 24-months after the index date was calculated as the proportion of patients who died of any reason during follow-up among all included CDI patients with corresponding 95% confidence intervals (CIs). To estimate all-cause mortality at 24 months, the analysis was restricted to patients included until December 31, 2017 (i.e., patients with a potential follow-up of at least 24 months). Excess mortality with 95% CIs was estimated by the difference in all-cause mortality rate between CDI and matched non-CDI patients during follow-up and reported per 100 patient-months.

HCRU and associated costs were estimated for the 12-month follow-up period using a payer perspective. The start of follow-up was set at index date for community-treated patients. For hospitalised CDI patients, follow-up started after discharge from the hospitalisation at index episode. HCRU was described overall and per patient. Costs were expressed in euros, inflated to 2020 rates. Tests for significance were not within the scope of the study. Total and average incremental costs per patient were estimated as the difference in the total and average costs, respectively, between CDI patients and the respective matched non-CDI patients.

Reporting of results followed the STrengthening the Reporting of OBservational studies in Epidemiology (STROBE) and Consolidated Health Economic Evaluation Reporting Standards 2022 (CHEERS) guidance [28, 29].

Results were presented for the population of CDI patients and stratified by the number of rCDIs (0, 1, 2, ≥ 3, ≥1) and for the non-CDI population. The reference group to estimate excess mortality and incremental costs of HCRU included only the respective matched non-CDI patients of each group of interest according to the number of rCDIs.

## Results

### Demographic and clinical characteristics

A total of 9,977 patients with an index CDI episode recorded between January 1, 2015 and December 31, 2018 were included in the study. Among these, 5,618 CDI patients were matched with 16,845 non-CDI patients.

Regarding the characteristics of the overall CDI patient population (*n* = 9,977), the median age was 77 years, and most were aged ≥ 65 years (*n* = 7,693; 77.11%). The proportion of patients ≥ 65 years was higher among patients with ≥ 1 rCDIs (*n* = 1,494; 82.91%) compared with those without rCDIs (*n* = 6,199; 75.83%). The proportion of women was slightly higher than men (*n* = 5,217; 52.29%) and increased among patients with 2 rCDIs (*n* = 179; 57.37%) and ≥ 3 rCDIs (*n* = 78; 58.21%). A median age-adjusted CCI score of 8 was calculated at index date, with little variation across subgroups. Oral antibiotics (*n* = 4,934; 49.45%) and proton pump inhibitors (PPIs) (*n* = 5,899; 59.13%) were the most common medications. The setting of infection of the index CDI episode was unknown for 43.66% of patients (*n* = 4,356). Of 56.34% of patients with an identified setting of infection (*n* = 5,621), 67.42% (*n* = 3,790) had a community-associated index CDI episode and 32.57% (*n* = 1,831) had a healthcare-associated index CDI episode. Most patients were treated in hospital settings (*n* = 8,816; 88.36%) (Table 2).

**Table 2:** Demographic and clinical characteristics of CDI and matched non-CDI patients (longitudinal cohort)

	All CDI patients	Non-recurrent CDI patients (0 rCDI)^a^	Patients with 1 rCDI^a^	Patients with 2 rCDIs^a^	Patients with ≥ 3 rCDIs^a^	Patients with ≥ 1 rCDIs^a^	All matched CDI patients	Matched non-CDI patients
**Total number of patients**								
N	9,977	8,175	1,356	312	134	1,802	5,618	16,845
**Age at index date (years)**								
Mean (SD)	73.35 (15.71)	72.87 (16.09)	75.03 (14.01)	76.77 (13.04)	77.28 (10.99)	75.50 (13.66)	71.87 (17.30)	71.80 (17.21)
Median; Q1 - Q3	77; 66.00–84.00	77; 65.00–84.00	78; 69.00–85.00	80; 72.00–85.00	80; 73.00–84.00	79; 70.00–85.00	77; 63.00–84.00	77; 63.00–84.00
Min; Max	18; 103	18; 103	18; 100	18; 96	21; 96	18; 100	18; 103	18; 104
Missing	0	0	0	0	0	0	0	0
**Age group at index, n (%)**								
18–64	2,284 (22.89%)	1,976 (24.17%)	248 (18.29%)	41 (13.14%)	19 (14.18%)	308 (17.09%)	1,516 (26.98%)	4,530 (26.89%)
≥ 65	7,693 (77.11%)	6,199 (75.83%)	1,108 (81.71%)	271 (86.86%)	115 (85.82%)	1,494 (82.91%)	4,102 (73.02%)	12,315 (73.11%)
**Gender, n (%)**								
Female	5,217 (52.29%)	4,257 (52.07%)	703 (51.84%)	179 (57.37%)	78 (58.21%)	960 (53.27%)	3,157 (56.19%)	9,468 (56.21%)
Male	4,760 (47.71%)	3,918 (47.93%)	653 (48.16%)	133 (42.63%)	56 (41.79%)	842 (46.73%)	2,461 (43.81%)	7,377 (43.79%)
**Charlson Comorbidity Index, age-adjusted, at index date**								
Mean (SD)	8.00 (3.95)	7.94 (4.00)	8.14 (3.76)	8.70 (3.45)	8.66 (3.15)	8.27 (3.67)	7.18 (4.07)	6.95 (4.10)
Median; Q1 - Q3	8; 5.00–11.00	8; 5.00–11.00	8; 6.00–11.00	9; 7.00–11.00	9; 6.00–11.00	8.5; 6.00–11.00	7; 4.00–10.00	7; 4.00–10.00
Min; Max	0; 23	0; 23	0; 21	0; 19	0; 16	0; 21	0; 21	0; 21
Missing	0	0	0	0	0	0	0	0
**Pre-index medical procedures, treatments and consultations, n (%)**								
Pre-index medications								
Antibiotics^b^	4,934 (49.45%)	4,075 (49.85%)	647 (47.71%)	154 (49.36%)	58 (43.28%)	859 (47.67%)	2,881 (51.28%)	8,639 (51.29%)
Laxatives	1,130 (11.33%)	937 (11.46%)	147 (10.84%)	31 (9.94%)	15 (11.19%)	193 (10.71%)	534 (9.51%)	1,113 (6.61%)
Proton pump inhibitors	5,899 (59.13%)	4,799 (58.70%)	819 (60.40%)	198 (63.46%)	83 (61.94%)	1,100 (61.04%)	3,078 (54.79%)	7,028 (41.72%)
H2-receptor antagonists	223 (2.24%)	188 (2.30%)	26 (1.92%)	8 (2.56%)	1 (0.75%)	35 (1.94%)	103 (1.83%)	317 (1.88%)
Selective immunosuppressants	144 (1.44%)	118 (1.44%)	23 (1.70%)	3 (0.96%)	0 (0.00%)	26 (1.44%)	64 (1.14%)	72 (0.43%)
TNF-α inhibitors	42 (0.42%)	39 (0.48%)	3 (0.22%)	0 (0.00%)	0 (0.00%)	3 (0.17%)	24 (0.43%)	38 (0.23%)
Interleukin inhibitors	9 (0.09%)	8 (0.10%)	0 (0.00%)	1 (0.32%)	0 (0.00%)	1 (0.06%)	5 (0.09%)	10 (0.06%)
Calcineurin inhibitors	115 (1.15%)	93 (1.14%)	19 (1.40%)	2 (0.64%)	1 (0.75%)	22 (1.22%)	48 (0.85%)	36 (0.21%)
Other immunosuppressants	187 (1.87%)	158 (1.93%)	25 (1.84%)	2 (0.64%)	2 (1.49%)	29 (1.61%)	102 (1.82%)	216 (1.28%)
Chemotherapies/ Antineoplastic agents	578 (5.79%)	483 (5.91%)	67 (4.94%)	18 (5.77%)	10 (7.46%)	95 (5.27%)	245 (4.36%)	700 (4.16%)
Monoclonal antibodies (Zinplava)	0 (0.00%)	0 (0.00%)	0 (0.00%)	0 (0.00%)	0 (0.00%)	0 (0.00%)	0 (0.00%)	0 (0.00%)
Pre-index medical procedures	1,168 (11.71%)	973 (11.90%)	139 (10.25%)	40 (12.82%)	16 (11.94%)	195 (10.82%)	483 (8.60%)	349 (2.07%)
**Setting of infection at index**								
Healthcare-associated	1,831 (18.35%)	1,390 (17.00%)	315 (23.23%)	88 (28.21%)	38 (28.36%)	441 (24.47%)	1,830 (32.57%)	5,490 (32.59%)
Community-associated	3,790 (37.99%)	3,164 (38.70%)	488 (35.99%)	97 (31.09%)	41 (30.60%)	626 (34.74%)	3,788 (67.43%)	11,355 (67.41%)
Unknown	4,356 (43.66%)	3,621 (44.29%)	553 (40.78%)	127 (40.71%)	55 (41.04%)	735 (40.79%)	0 (0.00%)	0 (0.00%)
**Setting of treatment at index**								
Hospitalised	8,816 (88.36%)	7,263 (88.84%)	1,178 (86.87%)	266 (85.26%)	109 (81.34%)	1,553 (86.18%)	4,626 (82.34%)	13,869 (82.33%)
Community-treated	1,161 (11.64%)	912 (11.16%)	178 (13.13%)	46 (14.74%)	25 (18.66%)	249 (13.82%)	992 (17.66%)	2,976 (17.67%)

CDI, *Clostridioides difficile* infection; Min, minimum; Max, maximum; Q1, 1st quartile; Q3, 3rd quartile; rCDI, recurrent CDI infection; SD, standard deviation; TNF-α, tumour necrosis factor-alpha

^a^Non-rCDI patients (0 rCDI), patients with 1 rCDI, patients with 2 rCDIs, patients with ≥ 3 rCDIs are subgroups of “All CDI patients”

^b^Antibiotics, including cephalosporins, fluoroquinolones, macrolides, penicillins with extended spectrum, clindamycin, and rifaximin

## All-cause mortality and excess mortality rate

All-cause mortality among CDI patients varied between 32.49% (*n* = 3,242) and 39.05% (*n* = 3,896) at 6-months and 12- months of follow-up, respectively. Among patients with a minimum follow-up of 24-months (*n* = 7,729), 47.60% (*n* = 3,679) died during this period. All-cause mortality remained mostly stable regardless of the number of rCDIs (Table 3).

**Table 3:** All-cause mortality at 6, 12, and 24 months of follow-up, stratified by number of rCDIs

Time	Statistical parameters	All CDI patients	Patients with no rCDI	Patients with 1 rCDI	Patients with 2 rCDIs	Patients with ≥ 3 rCDIs	Patients with ≥ 1 rCDIs
Death at 6 months	Number of patients at index	9,977	8,175	1,356	312	134	1,802
	Number of deaths	3,242	2,674	439	99	30	568
	6-month mortality rate (%)	32.49	32.71	32.37	31.73	22.39	31.52
	95% CI for proportion	[31.58; 33.41]	[31.69; 33.73]	[29.88; 34.87]	[26.57; 36.90]	[15.33; 29.45]	[29.38; 33.67]
Death at 12 months	Number of patients at index	9,977	8,175	1,356	312	134	1,802
	Number of deaths	3,896	3,203	522	125	46	693
	12-month mortality rate (%)	39.05	39.18	38.50	40.06	34.33	38.46
	95% CI for proportion	[38.09; 40.01]	[38.12; 40.24]	[35.91; 41.09]	[34.63; 45.50]	[26.29; 42.37]	[36.21; 40.70]
Death at 24 months	Number of patients at index^**a**^	7,729	6,317	1,060	248	104	1,412
	Number of deaths	3,679	3,000	506	125	48	679
	24-month mortality rate (%)	47.60	47.49	47.74	50.4	46.15	48.09
	95% CI for proportion	[46.49; 48.71]	[46.26; 48.72]	[44.73; 50.74]	[44.18; 56.63]	[36.57; 55.73]	[45.48; 50.69]

CDI: *Clostridioides difficile* infection; rCDI, recurrent CDI infection; CI, confidence interval

^a^Death at 24 months restricted to patients with a potential follow-up of at least 24 months

A mortality rate of 5.36, 3.54, and 2.50 deaths per 100 patient-months was estimated among matched CDI patients at 6, 12, and 24 months of follow-up, respectively. When compared to non-CDI patients, an excess mortality of 2.17, 1.35, and 0.94 deaths per 100 patient-months was estimated. Excess mortality was lower among patients with rCDIs. At 12-months of follow-up, excess mortality among non-recurrent CDI patients and those with ≥ 1 rCDI was 1.41 and 1.10 deaths per 100 patient-months, respectively. The low sample in patient with 2 and ≥ 3 rCDIs limits interpretation of results (Table 4).

**Table 4:** Excess mortality in non-CDI patients compared with CDI patients, stratified by number of rCDIs

Time	Statistical parameters	Non-CDI patients	All CDI patients	Patients without rCDI	Patients with 1 rCDI	Patients with 2 rCDIs	Patients with ≥ 3 rCDIs	Patients with ≥ 1 rCDIs
6-month mortality	Number of patients at index date	16,845	5,618	4,552	803	185	78	1,066
	Number of deaths	2,832	1,460	1,172	221	48	19	288
	Follow-up time in patient-months	88,691	27,241	21,914	3,946	963	417	5,327
	Mortality rate (/100 patient-months)	3.19	5.36	5.35	5.6	4.98	4.55	5.41
	Excess mortality (/100 patient-months)^a^	Ref.	2.17	2.26	2.06	1.20	0.32	1.78
	95% CI for excess mortality		1.87; 2.47	1.92; 2.59	1.25; 2.87	-0.38; 2.78	-2.04; 2.68	1.09; 2.46
12-month mortality	Number of deaths	3,680	1,795	1,444	257	65	29	351
	Follow-up time in patient-months	168,435	50,711	40,979	7,294	1,705	732	9,732
	Mortality rate (/100 patient-months)	2.18	3.54	3.52	3.52	3.81	3.96	3.61
	Excess mortality (/100 patient-months)^a^	Ref.	1.35	1.41	1.06	1.19	1.26	1.10
	95% CI for excess mortality		1.18; 1.53	1.21; 1.61	0.59; 1.54	0.16; 2.21	-0.34; 2.85	0.68; 1.52
24-month mortality	Number of deaths	4,658	2,216	1,769	330	82	35	447
	Follow-up time in patient-months	298,692	88,517	71,739	12,684	2,879	1,215	16,779
	Mortality rate (/100 patient-months)	1.56	2.50	2.47	2.60	2.85	2.88	2.66
	Excess mortality (/100 patient-months)^a^	Ref.	0.94	0.96	0.83	1.00	0.82	0.86
	95% CI for excess mortality		0.83; 1.06	0.84; 1.09	0.52; 1.14	0.33; 1.68	-0.24; 1.87	0.59; 1.13

CDI *Clostridioides difficile* infection; rCDI, recurrent CDI infection; CI, confidence interval

^a^The reference group to estimate excess mortality in CDI patients, overall and according to the number of ^r^CDI, was constituted by the respective matched non-CDI patients of each group of interest

### HCRU in patients with CDI and rCDI

HCRU was consistently higher among CDI patients compared with non-CDI patients and increased with the number of rCDIs. More than half of the CDI patients were hospitalised during follow-up (CDI: 54.59% [*n* = 3,067] vs. non-CDI: 41.02% [*n* = 6,910]). Furthermore, 78.42% (*n* = 836) of patients with ≥ 1 rCDI had a hospitalisation during follow-up, reaching 93.59% (*n* = 73) of patients with ≥ 3 rCDIs. Compared to non-CDI patients, CDI patients were more often hospitalised (median number of hospitalisations: 1 vs. 0), with a slightly higher length of stay (LOS) per hospitalisation (mean [SD] of 11.95 [12.72] days vs. 10.82 [12.34] days) and higher inpatient care days over follow-up (mean [SD] of 14.72 [26.77] days vs. 8.96 [20.96] days). Utilization of the following resources was also higher in CDI patients compared with non-CDI patients: pharmacological treatments (median of 9 vs. 8), medical devices (median of 2 vs. 1), and medical transportation (median of 2 vs. 1). The number of intensive care unit admissions, outpatient visits, and diagnostic tests was similar in both CDI and non-CDI patients. However, among CDI patients, utilization was higher among those with rCDIs (Table 5).

**Table 5:** HCRU in CDI and non-CDI patients during 12-months follow-up, stratified by number of rCDIs

	Non-CDI patients	All CDI patients	Patients without rCDI	Patients with 1 rCDI	Patients with 2 rCDIs	Patients with ≥ 3 rCDIs	Patients with ≥ 1 rCDIs
Total number of patients, *N*^a^	16,845	5,618	4,552	803	185	78	1,066
**Hospitalisations**							
Patients with hospital stays, n (%)	6,910 (41.02%)	3,067 (54.59%)	2,231 (49.01%)	609 (75.84%)	154 (83.24%)	73 (93.59%)	836 (78.42%)
Inpatient care days							
Mean (SD)	8.96 (20.96)	14.72 (26.77)	12.62 (24.75)	21.37 (33.27)	28.30 (29.86)	36.86 (27.14)	23.70 (32.58)
Median; Q1 - Q3	0; 0.00–9.00	3; 0.00–19.00	0; 0.00–15.00	12; 2.00–27.00	22; 8.00–38.00	34; 13.00–54.00	15; 3.00–32.00
Min; Max	0; 399	0; 348	0; 348	0; 330	0; 172	0; 121	0; 330
**Number of hospitalisations**							
N	13,938	6,922	4,952	1.303	423	244	1,970
Mean (SD)	0.83 (1.45)	1.23 (1.75)	1.09 (1.70)	1.62 (1.67)	2.29 (1.87)	3.13 (2.16)	1.85 (1.80)
Median; Q1 - Q3	0; 0.00–1.00	1; 0.00–2.00	0; 0.00–2.00	1; 1.00–2.00	2; 1.00–3.00	3; 2.00–4.00	1; 1.00–3.00
Min; Max	0; 26	0; 23	0; 23	0; 11	0; 10	0; 9	0; 11
**Length of stay per hospitalisation, days**							
Mean (SD)	10.82 (12.34)	11.95 (12.72)	11.60 (12.76)	13.17 (13.83)	12.38 (10.57)	11.78 (8.01)	12.83 (12.60)
Median; Q1 - Q3	7; 4.00–13.00	8; 4.00–15.00	8; 4.00–14.00	9; 5.00–16.00	9; 6.00–16.00	10; 6.00–16.00	9; 5.00–16.00
Min; Max	1; 198	1; 174	1; 174	1; 116	1; 87	1; 50	1; 116
**ICU admission, n (%)**							
Yes	412 (2.45%)	171 (3.04%)	118 (2.59%)	38 (4.73%)	9 (4.86%)	6 (7.69%)	53 (4.97%)
No	6,766 (40.17%)	3,021 (53.77%)	2,198 (48.29%)	597 (74.35%)	153 (82.70%)	73 (93.59%)	823 (77.20%)
**Outpatient visits**							
Patients with outpatient visits, n (%)	16,255 (96.50%)	5,224 (92.99%)	4,173 (91.67%)	789 (98.26%)	184 (99.46%)	78 (100.00%)	1,051 (98.59%)
Number of outpatient visits							
N	478,610	185,724	148,579	26,397	6,858	3,890	37,145
Mean (SD)	28.41 (24.98)	33.06 (36.01)	32.64 (36.50)	32.87 (31.04)	37.07 (31.27)	49.87 (56.07)	34.85 (33.79)
Median; Q1 - Q3	24; 12.00–39.00	25; 11.00–44.00	25; 10.00–43.00	27; 12.00–43.00	32; 18.00–47.00	39.5; 20.00–55.00	28; 13.00–45.00
Min; Max	0; 428	0; 406	0; 406	0; 250	0; 246	2; 364	0; 364
**Pharmacological treatment (outpatient)**							
Patients with pharmacological treatments, n (%)	15,403 (91.44%)	5,059 (90.05%)	4,020 (88.31%)	779 (97.01%)	183 (98.92%)	77 (98.72%)	1,039 (97.47%)
Number of pharmacological treatments							
N	147,101	57,376	45,162	8,699	2,456	1,059	12,214
Mean (SD)	8.73 (6.34)	10.21 (7.36)	9.92 (7.45)	10.83 (6.83)	13.28 (6.65)	13.58 (6.23)	11.46 (6.84)
Median; Q1 - Q3	8; 4.00–13.00	9; 5.00–15.00	9; 4.00–14.00	10; 6.00–15.00	13; 8.00–17.00	13; 9.00–17.00	11; 7.00–15.00
Min; Max	0; 46	0; 52	0; 52	0; 34	0; 37	0; 33	0; 37
**Medical procedures** ^**b**^							
Patients with medical procedures, n (%)	11,039 (65.53%)	3,136 (55.82%)	2,426 (53.30%)	507 (63.14%)	141 (76.22%)	62 (79.49%)	710 (66.60%)
Number of medical procedures							
N	47,548	14,675	11,265	2,488	629	293	3,410
Mean (SD)	2.82 (4.36)	2.61 (5.06)	2.47 (4.83)	3.10 (6.39)	3.40 (4.49)	3.76 (3.40)	3.20 (5.92)
Median; Q1 - Q3	1; 0.00–4.00	1; 0.00–3.00	1; 0.00–3.00	1; 0.00–4.00	2; 1.00–5.00	3; 1.00–6.00	1; 0.00–4.00
Min; Max	0; 72	0; 87	0; 87	0; 82	0; 27	0; 11	0; 82
**Diagnostic tests**							
Patients with diagnosis tests, n (%)	14,648 (86.96%)	4,834 (86.04%)	3,838 (84.31%)	741 (92.28%)	180 (97.30%)	75 (96.15%)	996 (93.43%)
Number of diagnosis tests							
N	88,927	34,347	27,115	5,296	1,278	658	7,232
Mean (SD)	5.28 (5.49)	6.11 (6.65)	5.96 (6.70)	6.60 (6.58)	6.91 (5.36)	8.44 (6.65)	6.78 (6.40)
Median; Q1 - Q3	4; 1.00–7.00	4; 2.00–8.00	4; 1.00–8.00	5; 2.00–9.00	6; 3.00–9.00	7; 4.00–12.00	5; 3.00–9.00
Min; Max	0; 78	0; 63	0; 63	0; 58	0; 34	0; 39	0; 58
**Medical devices** ^**c**^							
Patients with medical devices, n (%)	9,939 (59.00%)	3,716 (66.14%)	2,937 (64.52%)	556 (69.24%)	153 (82.70%)	70 (89.74%)	779 (73.08%)
Number of medical devices							
N	45,449	23,400	17,955	3,725	1,139	581	5,445
Mean (SD)	2.70 (4.33)	4.17 (6.12)	3.94 (6.03)	4.64 (6.40)	6.16 (6.20)	7.45 (6.40)	5.11 (6.42)
Median; Q1 - Q3	1; 0.00–3.00	2; 0.00–6.00	1; 0.00–5.00	2; 0.00–7.00	4; 1.00–10.00	6; 2.00–12.00	3; 0.00; 8.00
Min; Max	0; 65	0; 58	0; 58	0; 52	0; 32	0; 24	0; 52
**Medical transportation**							
Patients with medical transportation, n (%)	9,513 (56.47%)	3,885 (69.15%)	3,035 (66.67%)	621 (77.33%)	158 (85.41%)	71 (91.03%)	850 (79.74%)
**Number of medical transportations**							
N	34,938	20,188	15,005	3,588	1,015	580	5,183
Mean (SD)	2.07 (4.01)	3.59 (7.11)	3.30 (6.85)	4.47 (8.55)	5.49 (5.37)	7.44 (6.50)	4.86 (7.98)
Median; Q1 - Q3	1; 0.00–3.00	2; 0.00–5.00	1; 0.00–4.00	3; 1.00–5.00	4; 2.00–7.00	7; 4.00–10.00	3; 1.00–6.00
Min; Max	0; 122	0; 169	0; 169	0; 156	0; 43	0; 48	0; 156

CDI, *Clostridioides difficile* infection; ICU: intensive care unit; Min, minimum; Max, maximum; Q1, 1st quartile; Q3, 3rd quartile; rCDI, recurrent CDI infection; SD, standard deviation

^**a**^All HCRU captured for 12-month follow-up after index date

^b^Costs of medical procedures are not available in the BKK database.

^c^Medical devices include orthotic insert, walking aids, inhalation, incontinence aid, application aid (insulin injection, infusion pump), compression therapy, wheelchair

### HCRU costs in non-CDI and CDI groups

The total mean HCRU cost over follow-up was €12,893.56 per patient for CDI patients (vs. €8,786.54 for non-CDI patients) and increased with the experience of rCDIs up to €25,090.71for patients with ≥ 3 rCDIs. The respective total median HCRU cost was €6,050.00 per patient for CDI patients (vs. €3,462.00 for non-CDI patients) and increased with the experience of rCDIs up to €19,491.00 for patients with ≥ 3 rCDIs.

For each HCRU category, associated mean costs per patient were higher compared with non-CDI patients: hospitalisations (€6,945.77 vs. €4,410.42), outpatient visits (€1,551.45 vs. €1,170.62), pharmacological treatments (€2,340.15 vs. €1,968.95), diagnosis tests (€134.44 vs. €101.46), medical devices (€959.88 vs. €491.30), and medical transportation (€949.56 vs. €595.24).

The respective median costs per patient per HCRU category compared with non-CDI patients were the following: hospitalisations (€1,958.67 vs. €0), outpatient visits (€842.62 vs. €790.19), pharmacological treatments (€823.15 vs. €508.33), diagnosis tests (€51.22 vs. €32.01), medical devices (€155.77 vs. €73.69), and medical transportation (€321.45 vs. €104.74).

**Fig. 1:**
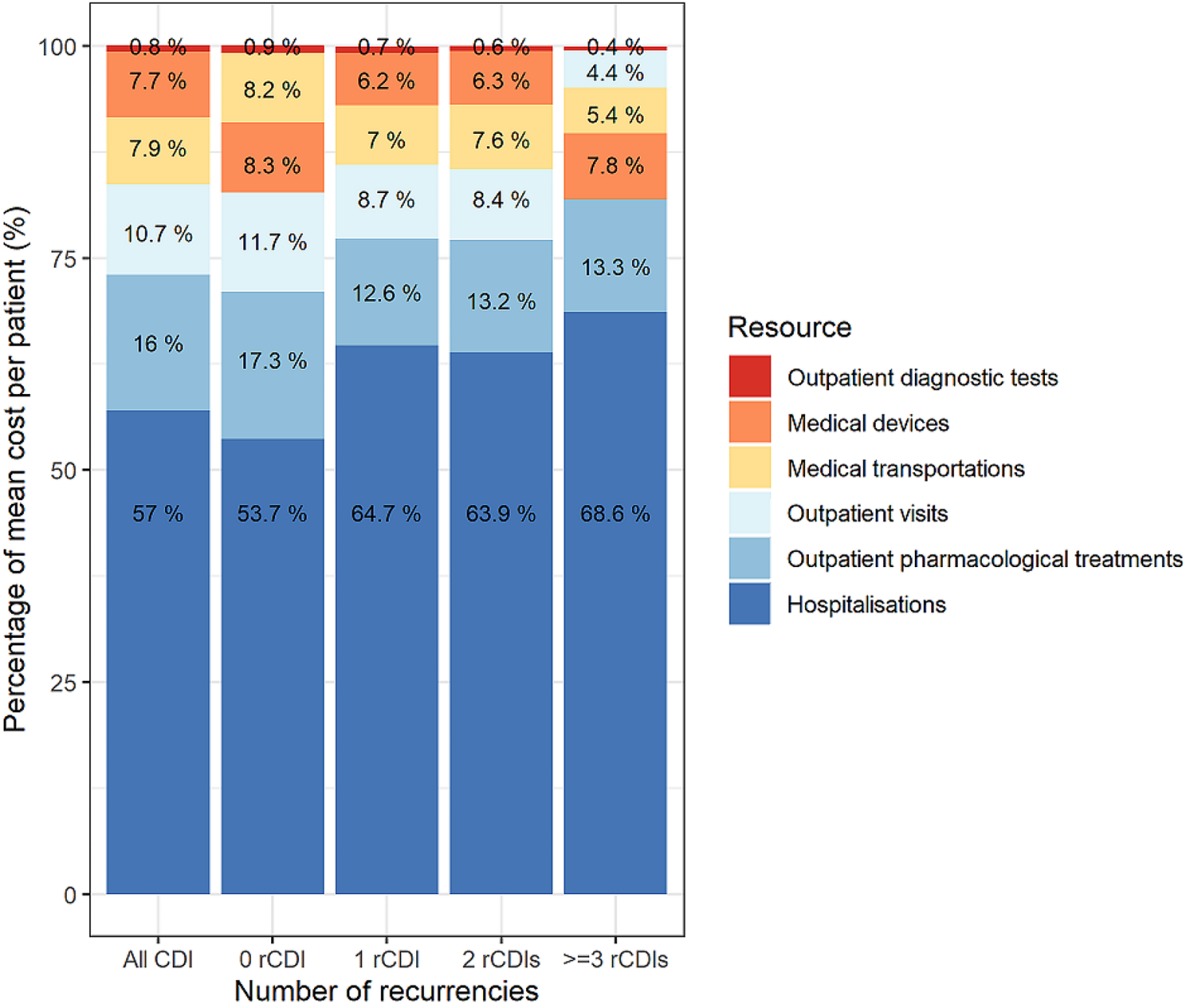
Mean HCRU costs per patient (%) stratified by type of resource and number of rCDIs

The costs increased alongside the number of rCDIs for all categories (Table 6). Hospitalisation costs were the major contributor to HCRU costs, varying from 53.70 to 68.60% of costs in patients without rCDI and with ≥ 3 rCDIs, respectively (Fig. 1).

**Table 6:** HCRU costs in CDI and non-CDI patients during 12-months follow-up stratified by number of rCDIs

	Non-CDI patients	All CDI patients	Patients without rCDI	Patients with 1 rCDI	Patients with 2 rCDIs	Patients with ≥ 3 rCDIs	Patients with ≥ 1 rCDIs
Total number of patients, *N*^a^	16,845	5,618	4,552	803	185	78	1,066
**Total costs of hospitalisations**							
Sum	74,293,526	39,021,360	28,275,052	7,320,715	2,204,854	1,220,739	10,746,308
Mean (SD)	4,410.42 (11,705.57)	6,945.77 (15,443.74)	6,211.57 (14,744.08)	9,116.71 (17,384.82)	11,918.13 (18,485.71)	15,650.50 (19,199.61)	10,080.96 (17,800.18)
Median; Q1 - Q3	0; 0.00–4,149.93	1,958.67; 0.00–7,732.67	0; 0.00–6,520.43	4,727.66; 899.18–10,425.54	7,612.80; 2,944.98–14,148.40	10,804.46; 5,585.87–20,290.10	5,593.31; 1,848.16–11,830.02
Min; Max	0; 252,725	0; 296,036	0; 296,036	0; 215,800	0; 159,713	0; 150,646	0; 215,800
**Total costs of outpatient visits**							
Sum	19,719,073	8,716,028	7,067,851	1,119,982	305,831	222,365	1,648,177
Mean (SD)	1,170.62 (2,239.12)	1,551.45 (3,930.69)	1,552.69 (3,937.66)	1,394.75 (2,967.72)	1,653.14 (3,839.18)	2,850.83 (9,039.43)	1,546.13 (3,902.64)
Median; Q1 - Q3	790.19; 399.74–1,333.42	842.62; 368.00–1,468.01	821.02; 333.40–1,462.11	864.41; 431.85–1,468.28	1,103.89; 634.18–1,577.01	1,145.41; 684.40–1,749.24	911.34; 462.49–1,511.65
Min; Max	0; 48,256	0; 72,835	0; 72,213	0; 34,912	0; 32,049	53; 72,835	0; 72,835
**Total costs of pharmacological treatments (outpatient)**							
Sum	33,166,920	13146,9,51	10,701,468	1,745,677	447,957	251,848	2,445,483
Mean (SD)	1,968.95 (7,405.30)	2,340.15 (5,914.38)	2,350.94 (6,152.12)	2,173.94 (5,189.58)	2,421.39 (3,029.57)	3,228.83 (3,340.45)	2,294.07 (4,769.82)
Median; Q1 - Q3	508.33; 121.13–1,489.39	823.15; 175.34–2,157.37	720.34; 139.04–2,062.82	985.52; 353.61–2,167.61	1,669.80; 796.89–3,025.72	1,976.64; 1,245.05–4,540.51	1,167.34; 466.25–2,416.88
Min; Max	0; 320,809	0; 163,197	0; 163,197	0; 89,893	0; 24,645	0; 18,988	0; 89,893
**Total costs of diagnosis tests**							
Sum	1,709,141	755,272	603,414	116,020	22,848	12,989	151,858
Mean (SD)	101.46 (178.64)	134.44 (233.40)	132.56 (232.42)	144.48 (259.32)	123.50 (140.87)	166.53 (174.97)	142.46 (237.47)
Median; Q1 - Q3	32.01; 0.00–129.45	51.22; 0.00–163.96	46.88; 0.00–161.49	65.97; 12.53–171.68	77.59; 24.69–155.49	111.39; 36.50–253.63	70.22; 14.81–175.73
Min; Max	0; 3,353	0; 2,958	0; 2,958	0; 2,934	0; 705	0; 779	0; 2,934
**Total costs of medical devices** ^**b**^							
Sum	8,275,906	5,392,579	4,409,000	615,262	240,368	127,949	983,578
Mean (SD)	491.30 (1,705.56)	959.88 (3,911.38)	968.59 (4,230.22)	766.20 (1,673.95)	1,299.28 (3,052.38)	1,640.37 (2,405.35)	922.68 (2,054.01)
Median; Q1 - Q3	73.69; 0.00–441.41	155.77; 0.00–782.19	127.46; 0.00–740.97	214.75; 0.00–753.24	506.14; 86.82–1,447.75	737.13; 191.20–2,032.44	295.82; 0.00–996.65
Min; Max	0; 137,552	0; 192,904	0; 192,904	0; 17,804	0; 35,362	0; 14,971	0; 35,362
**Total cost of medical transportations**							
Sum	10,026,793	5,334,627	4,124,180	843,045	233,228	134,174	1,210,447
Mean (SD)	595.24 (1,343.72)	949.56 (2,598.47)	906.01 (2,713.30)	1,049.87 (1,931.43)	1,260.69 (2,010.28)	1,720.18 (2,804.55)	1,135.50 (2,027.16)
Median; Q1 - Q3	104.74; 0.00–765.52	321.45; 0.00–1,025.71	252.22; 0.00–944.22	508.36; 59.84–1,266.29	818.21; 250.15–1,701.26	1,014.26; 465.20–2,015.92	577.36; 101.37–1,419.12
Min; Max	0; 36,831	0; 99,836	0; 99,836	0; 20,462	0; 21,462	0; 19,090	0; 21,462
**Total overall HCRU costs**							
Sum	148,009,254	72,436,022	55,292,480	11,736,836	3,449,631	1,957,075	17,143,543
Mean (SD)	8,786.54 (16,059.44)	12,893.56 (20,836.68)	12,146.85 (20,726.88)	14,616.23 (20,519.76)	18,646.66 (20,776.80)	25,090.71 (23,857.08)	16,082.12 (21,012.33)
Median; Q1 - Q3	3,462.00; 1,216.55–9,499.30	6,050.00; 1,619.06–15,352.13	4,956.00; 1,282.01–14,120.61	8,915.00; 4,109.31–16,389.21	13,797.00; 7,561.12–22,302.36	19,491.00; 9,873.86–29,239.82	10,060.00; 4,855.24–19,220.87
Min; Max	0; 362,674	0; 321,822	0; 321,822	31; 222,042	157; 168,106	1,400; 157,512	31; 222,042

CDI, *Clostridioides difficile* infection; Min, minimum; Max, maximum; Q1, 1st quartile; Q3, 3rd quartile; rCDI, recurrent CDI infection; SD, standard deviation

^**a**^ Total number of patients (regardless the use of each resource) used as denominator for all analyses. All HCRU captured for 12-month follow-up after index date. Costs are expressed in euros, inflated to 2020 rates

^b^ Medical devices include orthotic insert, walking aids, inhalation, incontinence aid, application aid (insulin injection, infusion pump), compression therapy, wheelchair. Total costs of medical procedures were not presented, since there are no individual values recorded in the BKK database

### Incremental HCRU costs in non-CDI and CDI groups

The highest incremental costs were observed for hospitalisations, with a mean incremental cost of €2,531.62 per patient in matched CDI compared with non-CDI patients. The mean incremental costs for matched CDI patients for outpatient visits, pharmacological treatments, diagnostic tests, medical devices, and medical transportation were €380.52, €369.49, €32.75, €468.77, and €353.99 per patient, respectively, compared with non-CDI patients. A total mean incremental cost of €4,101.15 was estimated per CDI patient. The mean incremental costs further increased with the number of rCDIs, with the highest total incremental cost of €13,291.78 recorded for patients with ≥ 3 rCDIs (Table 7).

**Table 7:** Incremental HCRU costs in CDI patients during 12-months follow-up stratified by number of rCDIs

Incremental costs during follow-up (€)	Non-CDI patients^a^	All CDI patients (matched cohort)	Non-rCDI patients (0 rCDI)	Patients with 1 rCDI	Patients with 2 rCDIs	Patients with ≥ 3 rCDIs	Patients with ≥ 1 rCDIs
Total number of patients, *N*	16,845	5,618	4,552	803	185	78	1,066
**Incremental costs of hospitalisations**							
Sum	Ref.	14,222,622	8,290,958	3,799,936	1,409,363	722,364	5,931,663
Mean (SD)	Ref.	2,531.62 (16,640.40)	1,821.39 (16,057.35)	4,732.17 (18,225.65)	7,618.18 (19,349.17)	9,261.08 (20,431.98)	5,564.41 (18,635.09)
**Incremental costs of outpatient visits**							
Sum	Ref.	2,137,756	1,734,505	188,986	95,724	118,542	403,251
Mean (SD)	Ref.	380.52 (4,104.82)	381.04 (4,114.01)	235.35 (3,214.47)	517.42 (3,993.73)	1,519.77 (9,027.18)	378.28 (4,067.28)
**Incremental costs of pharmacological treatments (outpatient)**							
Sum	Ref.	2,075,799	1,712,699	247,582	71,063	44,455	363,100
Mean (SD)	Ref.	369.49 (7,169.23)	376.25 (7,248.10)	308.32 (7,030.12)	384.13 (5,874.35)	569.93 (6,858.46)	340.62 (6,825.36)
**Incremental costs of diagnosis tests**							
Sum	Ref.	183,979	133,571	39,812	5,793	4,802	50,408
Mean (SD)	Ref.	32.75 (244.99)	29.34 (244.24)	49.58 (268.84)	31.32 (158.48)	61.57 (188.71)	47.29 (247.80)
**Incremental costs of medical devices**							
Sum	Ref.	2,633,573	2,203,230	207,626	139,218	83,499	430,343
Mean (SD)	Ref.	468.77 (4,035.98)	484.01 (4,359.15)	258.56 (1,806.48)	752.53 (3,151.74)	1,070.50 (2,528.95)	403.70 (2,169.26)
**Incremental costs of medical transportations**							
Sum	Ref.	1,988,379	1,433,702	355,927	122,402	76,348	554,677
Mean (SD)	Ref.	353.93 (2,665.55)	314.96 (2,781.14)	443.25 (1,966.70)	661.63 (2,054.19)	978.82 (3,160.15)	520.34 (2,094.55)
**Total incremental costs**							
Sum	Ref.	23,040,261	15,355,011	4,803,207	1,845,284	1,036,759	7,685,250
Mean (SD)	Ref.	4,101.15 (22,108.35)	3,373.24 (22,068.18)	5,981.58 (21,825.62)	9,974.51 (21,223.71)	13,291.78 (24,434.36)	7,209.43 (22,020.12)

CDI, *Clostridioides difficile* infection; rCDI, recurrent CDI infection; SD, standard deviation. All HCRU captured for 12-month follow-up after index date. Costs are expressed in euros, inflated to 2020 rates. Total costs of medical procedures were not presented, since there are no individual values recorded in the BKK database

^a^ Costs in CDI patients were compared with costs among the respective matched non-CDI patients according to the number of rCDIs. As matching of CDI patients and control non-CDI patients is usually not 1:1 (up to 3 non-CDI patients will be matched to a CDI patient), total costs for non-CDI patients were divided by the number of non-CDI patients per case before deriving the incremental difference

## Discussion

This observational retrospective cohort study estimated the mortality and economic burden of CDI and rCDI using real-world data from Germany between 2015 and 2019.

A total of 9,977 CDI patients were included in the study. At time of index CDI episode, patients had a median age of 77 years and were mostly treated in hospital settings. A substantial all-cause mortality rate was observed, with 32% of CDI patients dying within 6-months of follow-up, increasing to 39% and 48% of patients within 12- and 24-months of follow-up, respectively. All-cause mortality remained stable regardless the number of rCDIs, except among patients with ≥ 3 rCDIs that presented a lower all-cause mortality rate, particularly within 6-months of follow-up. When evaluating excess mortality among matched CDI patients in comparison to non-CDI patients with similar demographic and clinical characteristics, the highest excess mortality was observed among CDI patients within the first 6-months of follow-up (2.2 deaths in excess per 100 patient-months), with the difference gradually reducing over time and being minimal at 24-months of follow-up. Excess mortality appeared to be lower among patients with rCDIs, but the low sample size limits interpretation of results. A higher likelihood of patients dying at the index CDI episode or shortly after may provide a potential explanation for this observation.

Despite variations, the findings are mostly aligned with previous studies on the association of CDI with an increased risk of death, particularly among the elderly and patients treated in hospital settings [10, 12, 15].

A real-world study on healthcare-associated CDI treated in hospital settings in the United Kingdom showed an all-cause mortality rate of approximately 50% in CDI patients, compared to 30.2% among non-CDI patients within 12-months of hospital admission [12]. CDI patients had a significantly higher 12-month risk of death compared to non-CDI patients. When comparing CDI patients with and without recurrences, the 12-month all-cause mortality rate was similar (49.5% and 47.8%, respectively), but rCDI patients were found to be at a slightly higher risk of death than those with non-recurrent CDI [12].

In the US, two studies using Medicare claims data among the elderly have also shown the impact of CDI on patients’ risk of death [10, 15]. Olsen et al. found a 40.9% all-cause mortality rate in elderly CDI patients within 12-months of diagnosis, compared to 7.4% in control patients, and a CDI attributable mortality risk of 10.9% [15]. Similarly, Feuerstadt et al. estimated a 12-month all-cause mortality rate of 45.9% in elderly CDI patients. Among those with a first and second rCDI, 41% and 35% died within 12-months, respectively [10]. CDI-related deaths varied from 2.7% in patients with non-recurrent CDI to 25.4% in patients with ≥ 1 rCDI [10].

Using SHI data from a German region in 2012, Lübbert et al. found lower mortality estimates than the present study, with an all-cause mortality rate at 12-months of 21.6% in hospitalised CDI patients and 7.1% in CDI patients treated in outpatient settings [17]. In patients with a first and second rCDI, the all-cause mortality rate increased to 28.9% and 40% in hospitalised patients, and to 22.5% and 30.4%, in patients treated in outpatient settings, respectively [17]. The discrepancy in the results is likely explained by differences in sample size and study setting (i.e., data collected from a single region during a restricted time frame) [17].

Hospital data from public sources has shown a decline in CDI mortality in Germany between 2015 and 2019 (2,666 to 1,006 CDI-related deaths, respectively), with most deaths recorded among elderly patients [22]. However, the results are likely to be underestimated as data from death certificates includes only those where CDI was recorded as primary cause of death missing CDI-secondary diagnosis [22].

Despite not depicting trends over time, the results of this study complement this information on the mortality burden of CDI, since it captures deaths due to any cause among CDI patients treated in hospital and community settings at index date. Additionally, it allowed to estimate excess mortality among CDI patients in comparison to non-CDI patients matched on demographic and clinical characteristics.

Regarding HCRU and associated costs, CDI patients had a consistently higher use of resources than non-CDI patients. Additionally, a steady increase in the consumption of resources and associated costs alongside the number of rCDIs was observed. An overall mean cost of HCRU of €12,893 per CDI patient was obtained over 12-months of follow-up, compared to €8,786 among CDI patients and rising to €16,082 in patients with ≥ 1 rCDI. Incremental HCRU costs of €4,101 and €7,209 were estimated among matched CDI and ≥ 1 rCDI patients in comparison to non-CDI patients. Hospitalisations represented the biggest driver of costs among CDI patients. The mean hospitalisation costs for CDI patients were approximately 50% higher than those of non-CDI patients (€6,945 vs. €4,410, respectively), being more than twice as high in patients with ≥ 1 rCDIs (€10,080). Hospitalisations represented an incremental cost of €2,532 and €5,564 in matched CDI and ≥ 1 rCDI patients, compared to non-CDI patients.

Similar trends have been reported in earlier research despite substantial heterogeneity [20, 30, 31]. Prior studies from Germany with data collected between 2010 − 2012 have evaluated LOS and costs of CDI hospitalisations. Regional SHI data from has shown a median LOS of 9 days for patients hospitalised for CDI [17]. A study using data from 37 German hospitals reported a mean LOS of 11.9 days and mean hospitalisation cost of €4,132 per patient, representing an additional cost of €536 compared with controls [30]. Data from a tertiary care hospital showed an overall direct treatment cost per patient of €18,460 in CDI patients without rCDIs and of €73,900 in patients with ≥ 1 rCDIs, compared to €14,531 in controls [31].

This study presents a comprehensive overview of the mortality and economic burden of CDI in Germany. The use of real-world data from SHI using the BKK database is a major strength since it allowed to assemble a nationally representative cohort of CDI patients treated in both hospital and community settings. For matched cohort analysis to estimate excess mortality and incremental costs, the reference for each sub-group of matched CDI patients according to the number of rCDIs only included the respective non-CDI patients. This means that reference groups differ for each sub-group but ensures the similarities between CDI and non-CDI patients. This is particularly relevant since rCDI patients were found to be older and frailer (e.g., higher CCI score).

The following limitations must be acknowledged when interpreting the results. The use of claims data which are not primarily collected for research purposes, and chances of omission, miscoding, and misclassification cannot be ruled out. Diagnoses in German SHI data are only available on a quarterly basis. Thus, to ensure a good specificity for the identification of CDI patients, patients with gastrointestinal conditions other than CDI and those without prescription of an antibiotic indicated for CDI nor a test for the identification of bacterial toxin A or B were excluded, which may represent a source of bias. Moreover, due to the inability of the algorithm used to identify the setting of infection, which failed to classify 44% of index CDI episodes, only 5,618 out of 9,977 CDI patients (56.3%) were selected for the matched cohort analysis, also leading to a potential selection bias. However, it should be noted that matched CDI and non-CDI patients had very similar demographic and clinical characteristics. Caution is needed when interpreting the results on all-cause and excess mortality according to the number of rCDIs as the results may reflect immortal time bias since patients need to survive long enough to experience each rCDI episode [12]. Lastly, pharmacological treatments were only described in community settings. When administered in hospital settings these are not observable for description for HCRU. However, associated costs are accounted in hospitalisation stay invoices. Costs of medical procedures are also included within hospitalisation invoices but not available separately.

## Conclusions

CDI is associated with an increased risk of death and places a substantial burden on health systems due to higher use of HCRU, particularly hospitalisations. HCRU and subsequent costs are further exacerbated by each subsequent rCDI. The findings emphasize the need for therapeutic innovations to reduce the mortality and economic burden of CDI and rCDI.

### Electronic supplementary material

Below is the link to the electronic supplementary material.

Supplementary Material 1

## Data Availability

The data that support the findings of this study are available from statutory sickness funds but restrictions apply to the availability of these data, which were used under license for the current study, and are not publicly available. Data are available from the authors upon reasonable request, and with permission of data-providing sickness funds.

## Abbreviations

BKK: *Betriebskrankenkassen*
CDI: *Clostridioides difficile* infection
CI: Confidence Interval
HCRU: Healthcare Resource Usage
ICD-10: International Classification of Diseases Version 10
LOS: Length Of Stay
rCDI: Recurrent *Clostridioides Difficile* Infection
SD: Standard Deviation
SHI: Statutory Health Insurance
US: United States

## References

[1] Balsells E, Shi T, Leese C, Lyell I, Burrows J, Wiuff C, Campbell H, Kyaw MH, Nair H (2019). Global burden of Clostridium difficile infections: a systematic review and meta-analysis. J Glob Health.

[2] Fu Y, Luo Y, Grinspan AM (2021). Epidemiology of community-acquired and recurrent Clostridioides difficile infection. Th Adv Gastroenterol.

[3] Louie TJ, Miller MA, Mullane KM, Weiss K, Lentnek A, Golan Y, Gorbach S, Sears P, Shue YK (2011). Fidaxomicin versus Vancomycin for Clostridium difficile infection. N Engl J Med.

[4] Bouza E, Dryden M, Mohammed R (2008). Results of a phase III trial comparing tolevamer, Vancomycin and metronidazole in patients with Clostridium difficile-associated diarrhoea. Clin Microbiol Infect.

[5] Lowy I, Molrine DC, Leav BA, Blair BM, Baxter R, Gerding DN (2010). Treatment with monoclonal antibodies against Clostridium difficile toxins. N Engl J Med.

[6] McFarland LV, Elmer GW, Surawicz CM (2002). Breaking the cycle: treatment strategies for 163 cases of recurrent Clostridium difficile disease. Am J Gastroenterol.

[7] McFarland LV, Surawicz CM, Greenberg RN, Fekety R, Elmer GW, Moyer KA (1994). A randomized placebo-controlled trial of Saccharomyces boulardii in combination with standard antibiotics for Clostridium difficile disease. JAMA.

[8] Kelly CP (2012). Can we identify patients at high risk of recurrent Clostridium difficile infection?. Clin Microbiol Infect.

[9] Finn E, Andersson FL, Madin-Warburton M (2021). Burden of Clostridioides difficile infection (CDI) - a systematic review of the epidemiology of primary and recurrent CDI. BMC Infect Dis.

[10] Feuerstadt P, Nelson WW, Drozd EM, Dreyfus J, Dahdal DN, Wong AC, Mohammadi I, Teigland C, Amin A, Mortality (2022). Health Care Use, and costs of Clostridioides difficile infections in older adults. J Am Med Dir Assoc.

[11] Nanwa N, Sander B, Krahn M, Daneman N, Lu H, Austin PC (2017). A population-based matched cohort study examining the mortality and costs of patients with community-onset Clostridium difficile infection identified using emergency department visits and hospital admissions. PLoS ONE.

[12] Enoch DA, Murray-Thomas T, Adomakoh N, Dedman D, Georgopali A, Francis NA, Karas A (2020). Risk of complications and mortality following recurrent and non-recurrent Clostridioides difficile infection: a retrospective observational database study in England. J Hosp Infect.

[13] Banks A, Moore EK, Bishop J, Coia JE, Brown D, Mather H, Wiuff C (2018). Trends in mortality following Clostridium difficile infection in Scotland, 2010–2016: a retrospective cohort and case–control study. J Hosp Infect.

[14] Hensgens MPM, Goorhuis A, Dekkers OM, van Benthem BHB, Kuijper EJ (2013). All-cause and Disease-specific mortality in hospitalized patients with Clostridium difficile infection: a Multicenter Cohort Study. Clin Infect Dis.

[15] Olsen MA, Stwalley D, Demont C, Dubberke ER (2019). Clostridium difficile infection increases acute and chronic morbidity and mortality. Infect Control Hosp Epidemiol.

[16] Appaneal HJ, Caffrey AR, Beganovic M, Avramovic S, LaPlante KL (2018). Predictors of Mortality among a National Cohort of Veterans with recurrent Clostridium difficile infection. Open Forum Infect Dis.

[17] Lübbert C, Zimmermann L, Borchert J, Hörner B, Mutters R, Rodloff AC (2016). Epidemiology and recurrence rates of Clostridium difficile infections in Germany: a Secondary Data Analysis. Infect Dis Ther.

[18] Gabriel L, Beriot-Mathiot A (2014). Hospitalization stay and costs attributable to Clostridium difficile infection: a critical review. J Hosp Infect.

[19] Reigadas Ramírez E, Bouza ES (2018). Economic Burden of Clostridium difficile infection in European Countries. Adv Exp Med Biol.

[20] Wingen-Heimann SM, Davies K, Viprey VF, Davis G, Wilcox MH, Vehreschild M et al. Clostridioides difficile infection (CDI): a pan-european multi-center cost and resource utilization study, results from the combatting bacterial resistance in Europe CDI (COMBACTE-CDI). Clin Microbiol Infect. 2022. 10.1016/j.cmi.2022.12.019 36586512

[21] Jones AM, Kuijper EJ, Wilcox MH (2013). Clostridium difficile: a European perspective. J Infect.

[22] Brestrich G, Angulo FJ, Berger FK, Brosamle C, Hagel S, Leischker A (2023). Epidemiology of Clostridioides difficile infections in Germany, 2010–2019: a review from four public databases. Infect Dis Ther.

[23] Vehreschild M, Schreiber S, von Muller L, Epple HJ, Weinke T, Manthey C, Oh J, Wahler S, Stallmach A. Trends in the epidemiology of Clostridioides difficile infection in Germany. Infection. 2023:1–8. 10.1007/s15010-023-02044-5 PMC10170422 37162717

[24] Tricotel A, Antunes A, Wilk A, Dombrowski S, Rinta-Kokko H, Andersson FL, Ghosh S (2024). Epidemiological and clinical burden of Clostridioides difficile infections and recurrences between 2015–2019: the RECUR Germany study. BMC Infect Dis.

[25] Viprey VF, Granata G, Vendrik K, Davis GL, Petrosillo N, Kuijper EJ (2023). European survey on the current surveillance practices, management guidelines, treatment pathways and heterogeneity of testing of Clostridioides difficile, 2018–2019: results from the combatting bacterial resistance in Europe CDI (COMBACTE-CDI). J Hosp Infect.

[26] Feuerstadt P, Boules M, Stong L, Dahdal DN, Sacks NC, Lang K, Nelson WW (2021). Clinical complications in patients with primary and recurrent Clostridioides difficile infection: a real-world data analysis. SAGE Open Med.

[27] van Prehn J, Reigadas E, Vogelzang EH, Bouza E, Hristea A, Guery B (2021). European Society of Clinical Microbiology and Infectious diseases: 2021 update on the treatment guidance document for Clostridioides difficile infection in adults. Clin Microbiol Infect.

[28] Vandenbroucke JP, von Elm E, Altman DG, Gotzsche PC, Mulrow CD, Pocock SJ (2007). Strengthening the reporting of Observational studies in Epidemiology (STROBE): explanation and elaboration. PLoS Med.

[29] Husereau D, Drummond M, Augustovski F, de Bekker-Grob E, Briggs AH, Carswell C (2022). Consolidated Health Economic Evaluation Reporting Standards (CHEERS) 2022 explanation and elaboration: a report of the ISPOR CHEERS II Good practices Task Force. Value Health.

[30] Grube RF, Heinlein W, Scheffer H, Rathmayer M, Schepp W, Lohse AW, Stallmach A, Wilke MH, Lerch MM (2015). Economic burden of Clostridium difficile enterocolitis in German hospitals based on routine DRG data. Z Gastroenterol.

[31] Heimann SM, Vehreschild JJ, Cornely OA, Wisplinghoff H, Hallek M, Goldbrunner R (2015). Economic burden of Clostridium difficile associated diarrhoea: a cost-of-illness study from a German tertiary care hospital. Infection.

