# Supplementary material for: Estimating excess mortality and economic burden of Clostridioides difficile infections and recurrences during 2015–2019: the RECUR Germany study
Source: BMC Infect Dis. 2024 May 31;24:548. doi: 10.1186/s12879-024-09422-w (PMC11143700; doi:10.1186/s12879-024-09422-w)
Supplement: Supplementary file 1 — Supplementary Material 1 [file 12879_2024_9422_MOESM1_ESM.docx]

### **Supplementary Data**

# **Figures**

**
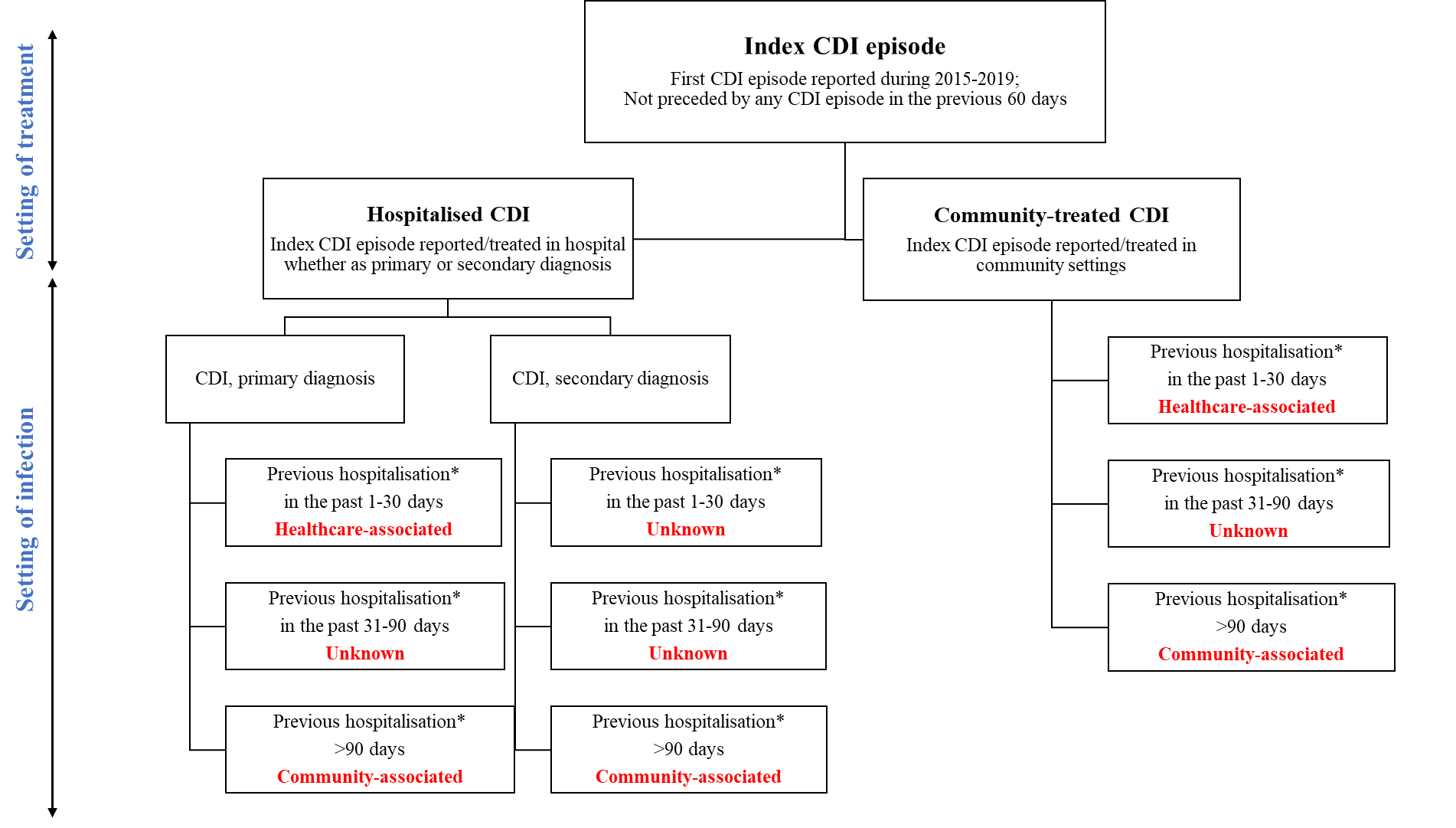
**

*Previous hospitalisation includes complete hospitalisation (>24 h or overnight stay, irrespective of cause) or transfer from a healthcare facility. CDI, *Clostridioides difficile* infection.

**Fig. S1** Classification of index CDI episodes by setting of treatment and infection.

# **Tables**

### **Table S1** Inclusion and exclusion criteria for patient selection

| **Inclusion criteria** | **Exclusion criteria** |
| --- | --- |
| Adult patient (≥18 years of age) with:   - A medical diagnosis indicating a *Clostridioides difficile* enterocolitis (ICD-10 code: A04.7) recorded in a hospital or community setting between 2015 and 2019 - The patient is observable in the database at least 12 months before the date of diagnosis. | 1. Patients with index CDI episode reported in community setting for whom the date of CDI onset cannot be approximated:  - Patients presenting with ≥1 gastrointestinal condition listed below during the same or the previous quarter of the CDI were excluded:   - A09 - Other gastroenteritis and colitis of infectious and unspecified origin   - K52 - Other noninfective gastroenteritis and colitis   - A48 - Other bacterial diseases not elsewhere classified   - K50 - Crohn disease   - K57 - Diverticular disease of intestine - Patients with no record of biological tests for identification of bacterial toxin A or B nor prescriptions of antibiotics indicated for CDI (non-topical metronidazole, or vancomycin or fidaxomicin) within the quarter of the diagnosis.  1. Patients without information on key demographics (e.g., year of birth). 2. Patients with a prior CDI episode within the previous 60 days (8 weeks) of the index CDI episode. 3. Patients with a CDI diagnosis recorded in the 6-month period prior to the index CDI episode |

CDI: *Clostridioides difficile* infection; ICD-10:International Classification of Diseases.
